# Clinical features and heteroplasmy in blood, urine and saliva in 34 Dutch families carrying the m.3243A > G mutation

**DOI:** 10.1007/s10545-012-9465-2

**Published:** 2012-03-09

**Authors:** Paul de Laat, Saskia Koene, Lambert P. W. J. van den Heuvel, Richard J. T. Rodenburg, Mirian C. H. Janssen, Jan A. M. Smeitink

**Affiliations:** 1 Department of Pediatrics, Nijmegen Centre for Mitochondrial Disorders, Radboud University Nijmegen Medical Centre, Huispost 804, Geert Grooteplein 10, 6500 HB PO BOX 9101, Nijmegen, The Netherlands; 2 Department of Internal Medicine, Nijmegen Centre for Mitochondrial Disorders, Radboud University Nijmegen Medical Centre, Nijmegen, The Netherlands

## Abstract

The m.3243A > G mutation has become known as the MELAS mutation. However, many other clinical phenotypes associated with this mutation have been described, most frequently being maternally inherited diabetes and deafness (MIDD). The m.3243A > G mutation, can be detected in virtually all tissues, however heteroplasmy differs between samples. Recent reports indicate, a preference to perform mutation analysis in urinary epithelial cells (UEC). To test this, and to study a correlation between the mutational load in different tissues with two mitochondrial scoring systems (NMDAS and NPMDS) we investigated 34 families carrying the m.3243A > G mutation. Heteroplasmy was determined in three non-invasively collected samples, namely leucocytes, UEC and buccal mucosa. We included 127 patients, of which 82 carried the m.3243A > G mutation. None of the children (n = 11) had specific complaints. In adults (n = 71), a median NMDAS score of 15 (IQR 10-24) was found. The most prevalent symptoms were hearing loss(48%), gastro-intestinal problems(42%), exercise intolerance(38%) and glucose intolerance(37%). Ten patients had neurologic involvement. Buccal mucosa had the best correlation with the NMDAS in all adults (r = 0.437,p < 0.001), whereas UEC had the strongest correlation with the NMDAS in severely affected patients (r = 0.593,p = 0.002). Heteroplasmy declined significantly with increasing age in all three samples (leucocytes r = -0.705 (p < 0.001), UEC r = -0.374(p = 0.001), buccal mucosa r = -0.460(p < 0.001). In our cohort of 82 patients, the m.3243A > G mutation causes a wide variety of signs and symptoms, MIDD being far more prevalent than MELAS. Looking at the characteristics of the three non-invasively available tissues for testing heteroplasmy we confirm that UEC are the preferred sample to test.

## Introduction

Mitochondria are responsible for production of adenosine triphosphate (ATP), through oxidative phosphorylation (OXPHOS). Mitochondrial dysfunction can result from mutations in either nuclear DNA (nDNA) or mitochondrial DNA (mtDNA).

The incidence of congenital mitochondrial disorders based on an OXPHOS defect is at least 1:8500 of all live births (Chinnery and Turnbull 2001).

Mitochondrial DNA encodes 37 genes, 22 code for tRNA’s, two for rRNA’s and 13 code for subunits of the OXPHOS complexes I, III, IV and V.

Two important features of mitochondrial mutations are; A) maternal inheritance, it can be assumed that all relatives from the maternal line are (dormant) carriers of the mutation. This applies only if there is not a *de novo* mutation, and B) heteroplasmy, human cells contain over 100 – 1000 mitochondria each, every mitochondrion contains 1-10 copies of mtDNA. When all mtDNA copies are mutated, there is a homoplasmic mutation. When however not all mtDNA copies are mutated, there is a variation in mutation load and the mutation is called heteroplasmic (Cree et al. 2009). Heteroplasmy can vary between different tissues of one patient and between one tissue in one patient in time.(Frederiksen et al. 2006) This variation in heteroplasmy between tissues complicates the demonstration of a relationship between the severity of the disease in a non accessible organ (e.g., the heart or the brain) and the degree of heteroplasmy in blood, urine or buccal mucosa.

The acronym MELAS was first used in 1984 by Pavlakis et al to describe a group of patients with Mitochondrial myopathy, Encephalopathy, Lactic Acidosis and Stroke-like episodes(Pavlakis et al. 1984). In 1990 the adenine to guanine transition at position 3243 of mitochondrial DNA (m.3243A > G) in the *MT-TL1* gene encoding tRNA^LEU(UUR)^ was found as molecular basis for this disease (Goto et al. 1990; Kobayashi et al. 1990). A mutation in tRNA commonly causes a combined defect of the mtDNA encoded OXPHOS complexes. tRNA^LEU(UUR)^ is partially (∼12%) responsible for the incorporation of leucine into mitochondrial DNA encoded proteins, tRNA^LEU(CUN)^ is responsible for the other part (∼88%) (Wittenhagen and Kelley 2003). It is proposed that because of the minimal role of tRNA^LEU(UUR)^ in incorporation of leucine, a mutation in the tRNA^LEU(UUR)^ is less lethal and therefore more frequently detected (Wittenhagen and Kelley 2003). The m.3243A > G mutation in the *MT-TL1* gene is the most common cause of MELAS syndrome, therefore the mutation is also known as the MELAS mutation. Later, more and more phenotypic expressions of the m.3243A > G mutation were found, including maternally inherited diabetes deafness (MIDD)(van den Ouweland et al. 1992), hypertrophic cardiomyopathy(Lev et al. 2004), macular dystrophy(Michaelides et al. 2008), focal segmental glomeruloslerosis (FSGS) (Lowik et al. 2005), myoclonic epilepsy with ragged-red fibers (MERRF)(Durand-Dubief et al. 2004) and oligosymptomatic variants of the acronym MELAS (Ma et al. 2010). Epidemiologic studies show a m.3243A > G mutation prevalence of 7.59/100.000 in the population of North-East England (Chinnery et al. 2000), 16.3/100.000 in the population of Northern Finland (Majamaa et al. 1998) and up to 236/100.000 in the population of Australia (Manwaring et al. 2007). These studies did not give a complete insight in the phenotypic expression of the m.3243A > G mutation in the subjects examined. The high mutation prevalence in the most recent study indicates a high number of undiagnosed carriers of the m.3243A > G mutation.

Since mitochondria and mtDNA are present in almost all tissues, heteroplasmy can theoretically be assessed in virtually every tissue. Two problems arise when testing heteroplasmy: the convenience of obtaining the sample and the differences in heteroplasmy levels between samples. For example, invasively obtained muscle tissue heteroplasmy usually gives a higher and more consistent level than conveniently attained blood, in the last case possibly leading to false-negative results. Recent studies showed a superiority of urine over blood as preferred non-invasive tissue for mutation analysis (Frederiksen et al. 2006; Ma et al. 2009; Marotta et al. 2009). The relationship between mutation load and clinical phenotypes has been a subject of research for many years (Chinnery et al. 1997). Whittaker et al. (2009) and Ma et al. (2009) recently showed a relationship between heteroplasmy levels in urinary epithelia and clinical symptoms in a small number of patients.

Some of the many questions regarding the m.3243A > G mutation that we have include: Are all mutation carriers symptomatic? Should these carriers undergo screening for frequent and preventable symptoms such as cardiomyopathy, glucose intolerance, nephropathy or macular dystrophy? How should female carriers be counseled regarding fertility questions?

In this study, we clinically evaluated 34 Dutch families, probands and maternal relatives, carrying the m.3243A > G mutation. We provide data about the whole phenotypic spectrum of m.3243A > G mutation as well as information regarding the correlation between the level of heteroplasmy in different samples and the clinical severity of the disease.

## Patients and methods

### Patients

All probands are patients of the Nijmegen Center for Mitochondrial Disorders at the Radboud University Nijmegen Medical Centre, The Netherlands, diagnosed with the m.3243A > G mutation in muscle or blood. All patients and maternal relatives were recruited by a letter, in which they were invited to participate in this study. This study was approved by the ethics committee of the Nijmegen-Arnhem region, The Netherlands. Written informed consent according to the Helsinki agreement was obtained from all parents and patients ≥12 years.

### Newcastle mitochondrial disease scales

All patients were invited for a single visit to our out-patient clinic. Adult patients (>18 yr) were scored using the Newcastle Mitochondrial Disease Adult Scale (NMDAS)(Schaefer et al. 2006), pediatric patients (<18 yr) were scored using the Newcastle Pediatric Mitochondrial Disease Scale (NPMDS)(Phoenix et al. 2006). The NMDAS and NPMDS constitute a validated method to monitor the clinical expression of mitochondrial disease and to follow-up the course of disease in time. The NMDAS and NPMDS consist of the following four sections. 1) Current function, gives insight into the general functioning of patients in the past four weeks. 2) System-specific involvement, uses a clinical history supplemented by specific information to gain insight in the functioning of individual organ-systems. 3) Current clinical assessment, a general and neurological clinical examination, gives insight in the current functional status of the patient. This section includes three cognition tests. We were able to use the English symbol test, since it does not use language. For the reading test we used a Dutch equivalent test. In absence of a Dutch equivalent test for the Speed of Comprehension we could not use this test. We scored the cognition based on the symbol test and reading test only.

Section 1 to 3 of the NMDAS consists respectively of ten, nine and ten questions, which can be scored from 0 (no involvement) to 5 (severe involvement). For section 4, Quality of life, we used a Dutch translation of the SF-12v2 quality of life test. A score from 0 to 70 for mental and physical health, where 50 is the population mean, is obtained from this quality of life score. The NPMDS exist respectively of seven, ten and nine questions, which can be scored from 0 (no involvement) to 3 (severe involvement). Section 4: Quality of life, uses a specially designed quality of life questionnaire for children. These do not have a reference score, so we can only use them to show a difference in time, therefore they are not mentioned in this report. Intra-observer and inter-observer variability was shown to be low in both the NMDAS and the NPMDS (Phoenix et al. 2006; Schaefer et al. 2006). All patients were scored independently by the same two investigators (PdL, SK), after which consensus was reached. The consensus score is used in the results of the study.

### m.3243A > G mutation analysis

At the same visit during which the NMDAS or NPMDS was scored, blood, saliva and urine was collected for heteroplasmy analysis. DNA was isolated from peripheral blood leukocytes using a salting-out method. The urine sediments and buccal swab samples were centrifuged for 10 min at 3000 r.p.m., the pellet was washed with phosphate-buffered saline. DNA was extracted using a commercially available DNA isolation kit (PuregeneTM DNA isolation kit; Gentra Systems, MN).

DNA samples were analyzed quantitatively using PyrosequencingTM technology (Pyrosequencing, Upsala, Sweden). Pyrosequencing was performed according to the protocol of the manufacturer. PCR of a mtDNA fragment containing the 3243 position was performed using the following primers: universal primer (biotinylated), 50-GGGACACCGCTGATCGTTTA-30; forward primer, 50-GACGGGACACCGCTGATCGTTTACAACTTAGTA TTATACCCACAC-30; and reverse primer, 50-ATTAGAATGGGTACAATGAGGA-30. PCR was carried out in a 50 ml volume containing 0.02 mM forward primer, 0.2 mM reverse primer and 0.2 mM of the biotinylated universal primer. PCR conditions were 92°C for 30 s, 55°C for 30 s, 72°C for 30 s, for a total of 40 cycles. Single-stranded template DNA, which in the present assay is the forward strand of the fragment, was purified using streptavidin-coated Sepharose beads. The actual pyrosequencing was performed on the PSQ96 platform using sequence primer 50-TATGCGATTACCGGGC-30. In a pyrosequence reaction, the four different deoxynucleotide triphosphates (dNTPs) are added separately one after the other. The incorporation of dNTP is accompanied by release of pyrophosphate (PPi). This PPi is involved in a light-producing reaction of which the amount of light produced is proportional to the number of nucleotides incorporated. The light is detected by a charge coupled device camera and seen as a peak in a pyrogram. Apyrase, a nucleotide-degrading enzyme, continuously degrades ATP involved in the light-producing reaction, and unincorporates dNTPs. This switches off light production and regenerates the reaction solution. Because the forward strand is used as template in the sequencing reaction, the change detected in the present assay concerns a T to C exchange. In fact, the amount of dTTP and dCTP incorporated at position 3243 during the sequencing reaction was determined in this way and from this the percentage of heteroplasmy was calculated. The pyrosequence reaction of the m.3234A > G mutation has a precision of 1.5%. The mutation is detected from a heteroplasmy level of 5%. The detection limit for the MELAS mutation (m.3243A > G) was determined by serial dilution of a sample containing this mutation with wild type mtDNA.

### Statistics

We used descriptive statistics to present patient characteristics and the results of the NMDAS and NPMDS. All data are presented as a median with interquartile ranges (IQR, 25th and 75th percentiles). Non-parametric tests were used if the data did not reflect a Gaussian distribution. Spearman’s rho correlation coefficient was used to evaluate the relationship between the clinical scores with the heteroplasmy in the different tissues. Pearson’s correlation was used to correlate heteroplasmy in different levels to each other and to age. A t-test was used to compare the quality of life of the m.3243A > G mutation carriers to reference values .

## Results

### Patient characteristics

Included in the study were 127 individuals from 34 families. The cohort consists of 24 probands. Fourty-eight family members were related in the first degree to a proband, 28 in the second degree, 18 in the third degree and nine in the fourth degree. The age of the patients ranged from two months to 80 years, median 39 years (IQR: 25 – 54 yr). Forty-two patients were male and 85 female (ratio 1 : 2.02). In 82 individuals the m.3243A > G mutation was detected in at least one of the samples. In 45 family members the mutation was not detected.

### Children

Eleven children (4 male, 7 female) carrying the m.3243A > G mutation were scored using the NPMDS. Two boys were probands, the other nine patients were maternal family members. The median age was 6 years (IQR 5.5-8.5 yrs). Five children scored zero points on the NPMDAS, two children scored one point, three children scored two points and one child scored four points. The main complaints were a delay in development (in five children) in early childhood, which had been caught up by the time of investigation. One girl attended special school for learning difficulties. One boy had communicational problems both in his native language (Turkish) and in Dutch. Heteroplasmy in UEC in these children ranged from 8-93% and had a median level of 59% (IQR : 31.5 – 77%).

### Adults

Seventy-one adult carriers (25 male, 46 female) of the m.3243A > G mutation were scored using the NMDAS. The median age was 45 years (IQR: 34-55 yr).

They had a median clinical score of: 4 (IQR: 1-7) on section 1; 4 (IQR 1-8) on section 2; and 3 (IQR 0-6) on section 3. The total median score of section 1-3 was 15 (IQR 10-24).

**Fig. 1:**
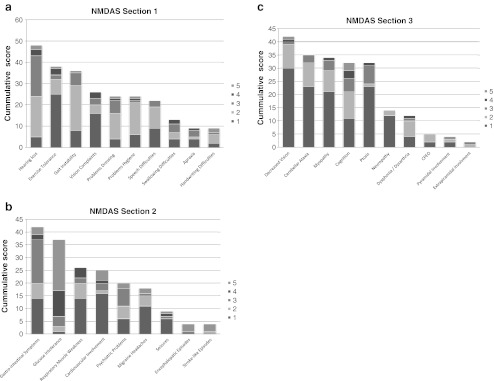
**a**-**c**: All clinical features scored by the NMDAS. Results of the Newcastle Mitochondrial Disease Adult Scale (NMDAS) are shown divided in the three sections of the scale, for all adult carriers of the m.3243A > G mutaion (n = 71). **a**) Section 1: Current function; **b**) Section 2: System-specific involvement; C) Section 3: Current clinical assessment

Table 1 presents the symptoms sorted by prevalence. A complete overview of all results can be found in Figs. 1a-c.

**Table 1:** Signs and symptoms of the m.3243A > G mutation scored by the NMDAS, sorted by prevalence (n = 71)

Signs & symptoms	Prevalence (%)
Hearing loss	48
Gastro-intestinal symptoms	42
Decreased vision	42
Exercise intolerance	38
Glucose intolerance	37
Gait instability	36
Cerebellar ataxia	35
Myopathy	34
Cognition < 33th percentile	32
Ptosis	32
Cardiovascular involvement	31
Vision complaints	26
Respiratory muscle weakness	26
Problems dressing	24
Problems hygiene	24
Speech difficulties	22
Psychiatric problems	20
Migraine headaches	18
Neuropathy	14
Swallowing difficulties	13
Dysphonia / dysarthria	12
Apraxia	9
Handwriting difficulties	9
Seizures	9
CPEO	5
Encephalopatic episodes	4
Stroke-like episodes	4
Pyramidal involvement	4
Extrapiramidal involvement	2

#### Summary of the most important findings

##### Hearing

Fourty-eight carriers (67.6%) had hearing difficulties, 24 carriers experienced mild deafness, whereas another 19 carriers had moderate deafness (not fully corrected with hearing aid), three carriers had severe deafness (poor even with hearing aid) and two carriers had end stage deafness requiring a cochlear implant.

Exercise Tolerance: 38 carriers (53.5%) had a compromised exercise tolerance. Twenty-five were only limited on inclines or stairs, whereas the other carriers were also limited on flat services.

Gait stability and cerebellar ataxia: 36 carriers (50.7%) reported difficulties maintaining their stability, 35 carriers (49.3%) had difficulties with the heel-toe test at the clinical assessment. Eight carriers reported difficulties on uneven grounds and 21 carriers reported occasional balance problems when walking. Six carriers reported occasional falls because of balance problems, one patient was unable to walk unsupported. On clinical assessment 23 carriers had a hesitant heel-toe test, nine were unable to maintain heel-toe walking and three carriers were unable to walk heel-toe.

##### Gastro-intestinal symptoms

Fourty-two carriers (59.2%) reported gastro-intestinal symptoms. Fourteen had mild constipation, six had occasional symptoms of irritable bowel. Seventeen carriers had severe constipation (requiring daily medication) or severe irritable bowel complaints, in five carriers gastro-intestinal symptoms were that severe they needed hospital admission, three of them underwent surgical procedures for gastro-intestinal dysmotility.

##### Diabetes mellitus

Thirty-seven carriers (52.1%) had a compromised glucose tolerance, for which ten were treated with tablets (non insulin depending diabetes mellitus) and 20 required insulin treatment (insulin depending diabetes mellitus). Four patient were on a diet and two carriers received no treatment for impaired glucose tolerance, one patient reported diabetes gravidarum.

##### Ptosis

Thirty-two carriers (45.1%) had a ptosis, mild ptosis (not obscuring either pupil) occurred in 23 carriers, moderate ptosis occurred in eight carriers, whereas one patient had a severe ptosis (obscuring bilateral >1/3 of pupils).

##### Myopathy

Thirty-four carriers (47.9%) had reduced muscle strength. Twenty-one carriers had minimal muscle weakness in hip flexion or shoulder abduction only (MRC 4+/5). Eight carriers had mild proximal muscle weakness (MRC 4/5) and four carriers had moderate muscle weakness (MRC 4) with difficulty to rise from 90 degree squat. One patient was unable to rise from 90 degree squat.

##### Cardiomyopathy

Twenty-five carriers (38.5%) had cardiac involvement. Sixteen had asymptomatic changes on electrocardiogram, mainly repolarization disturbances. One had asymptomatic left ventricular hypertrophy (LVH). Seven had cardiomyopathy of which four had a ejection fraction of less than 30%. One patient had a pacemaker for a complete AV-block.

##### Epilepsy

A history of seizures was present in nine carriers, of which three had had seizures in the past year. Stroke-like episodes: a history of stroke-like episodes was present in four carriers, of which three had Stroke-like Episodes in the past year.

##### Further neurologic examination

Twelve carriers had subtle sensory symptoms or areflexia (in knee and elbow reflexes). Four carriers showed symptoms of pyramidal involvement and two carriers showed symptoms of extrapyramidal involvement.

**Fig. 2:**
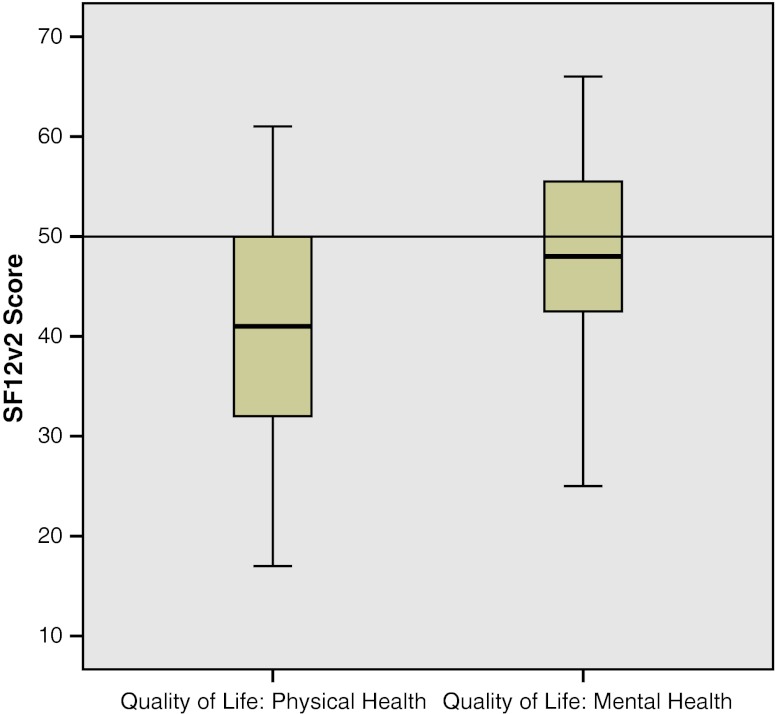
Quality of life Section 4 of the NMDAS is a QoL test, divided in QoL_P_ and QoL_M_. All adult carriers of the m.3243A > G mutation are shown (n = 71). The line at 50 shows the US average of the test. QoL_P_ is significantly lower than the US average

Section 4 of the NMDAS scores quality of life (QoL). It distinguishes quality of physical health (QoL_P_) and quality of mental health (QoL_M_). QoL_P_ had a range of 17 to 61, with a median of 41 (IQR 32-50). QoL_M_ had a range of 25 to 66, with a median of 48 (IQR 42-48) (Fig. 2).

Using a t-test a significant (P < 0.001) lower QoL_P_ was found in patients carrying the m.3243A > G mutation against the test average of 50. No difference in QoL_M_ in carriers against the test average could be found (p = 0.055). Comparing quality of life in carriers to non-carriers, there was a significant lower QoL_P_ in carriers (P < 0.001) and no difference in QoL_M_ (p = 0.395). The QoL_P_ was significantly negatively correlated to the NMDAS (r = -0.565 p > 0.001). There was no correlation between QoL and the heteroplasmy in any of the samples.

### Heteroplasmy levels

**Fig. 3:**
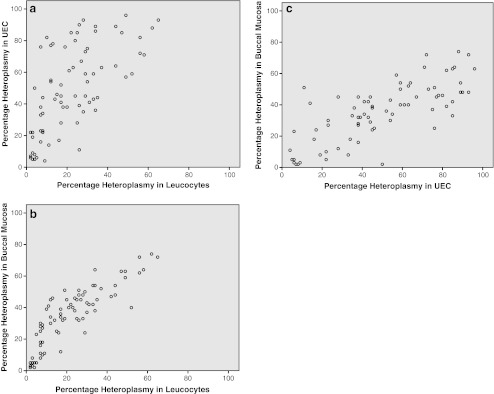
**a**-**c**: Heteroplasmy levels in all patients carrying the m.3243A > G mutation (n = 82) in three different samples (leucocytes, UEC and buccal mucosa) are correlated

**Fig. 4:**
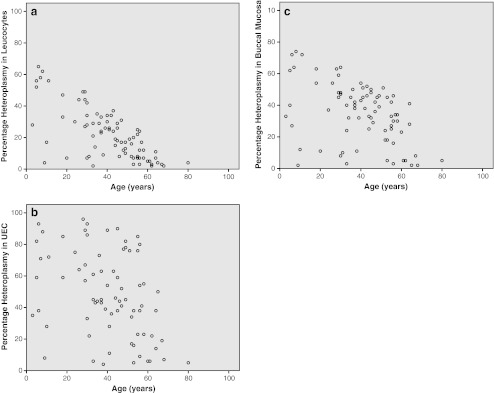
**a**-**c**: Heteroplasmy vs age heteroplasmy levels in all patients carrying the m.3243A > G mutation (n = 82) show a negative correlation to age in leucocytes, UEC and buccal saliva

Heteroplasmy levels in leucocytes were determined in 126 individuals (one child refused the blood draw), in UEC in 121 individuals (insufficient material was received at the laboratory in six individuals) and in buccal mucosa in all 127 individuals. Eighty-two individuals had a heteroplasmy level of ≥5% in at least one of the samples. In the carriers, the mean heteroplasmy in leucocytes was 22% (range 2-65%), in UEC 48% (range 4-96%) and 35% (range 2-74%) in buccal mucosa. Despite these differences between the samples there were strong pair wise correlations between the samples (r = 0.657-0.854 p < 0.001 / see Figs. 3a-c). A negative correlation between the level of heteroplasmy and age was present in all samples , for leucocytes r = -0.678 (p < 0.001), for UEC r = -0.337 (p = 0.003), and for buccal mucosa r = -0.432 (p < 0.001) (see Figs. 4a-c).

The heteroplasmy level in UEC was below the detection limit in one (1%) carrier that did have a detectable mutation load in buccal mucosa and leucocytes. Heteroplasmy levels in buccal mucosa were below the detection limit in seven (9%) carriers that did have a detectable mutation load in UEC. Heteroplasmy levels in leucocytes were below the detection limit in 12 (15%) carriers that did have a detectable mutation load in UEC.

**Fig. 5:**
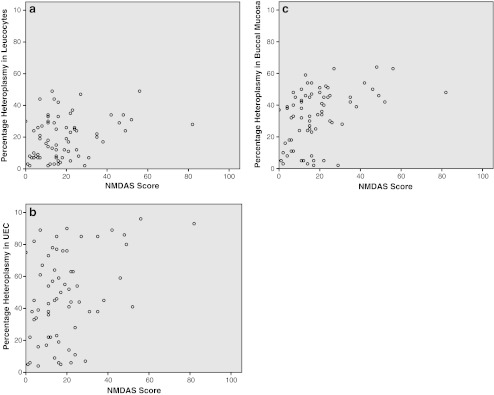
**a**-**c**: Heteroplasmy vs NMDAS score Heteroplasmy levels in leucocytes, UEC and buccal saliva in all adult patients carrying the m.3243A > G mutation (n = 71) show a correlation to the score on the NMDAS

A correlation between clinical condition of the patient and the level of heteroplasmy could not be made using the NPMDS in children, because of the limited number of pediatric carriers included in the study. In adults, there was a correlation between the score on the NMDAS and the heteroplasmy, in leucocytes r = 0.254 (p = 0.032), in UEC r = 0.294 (p = 0.016), and in buccal mucosa r = 0.427 (p < 0.001) (See figs. 5a-c. Patients with a NMDAS of more than 20 (n = 25), have a better correlation between the clinical scores and the heteroplasmy levels in leucocytes (r = 0.366 (p = 0.072)), and UEC (r = 0.530 (p = 0.008)).

## Discussion

### Dormant carrier

Following the results of this study, we propose to introduce a new term in the mitochondrial genetic nomenclature: dormant carrier. The term “dormant carrier” is used to indicate that a patient that has a mitochondrial mutation, but has no obvious clinical symptoms of the mutation (yet). We have chosen this new term because we experienced that the nomenclature in mitochondrial inheritance has an overlap with nomenclature in Mendelian inheritance, which causes confusion in providing patient information. In the Mendelian nomenclature, a carrier is a patient that has a recessive mutation on one allele, and is never going to be a patient. On the contrary, a dormant carrier of a mitochondrial mutation may at some point become symptomatic (awake), depending on heteroplasmy levels and unknown other factors. In patient counseling and preventive medicine this makes a big difference.

### NMDAS and NPMDS

Since the Newcastle scores consists of subjective questions regarding the personal opinion of the patient on daily functioning (Section 1) and the interpretation of the findings at physical examination (Section 3), all patients were scored separately by two investigators.

Only ten patients aged under 18 were included in the study. All ten children had no or only minor complaints. However, we did include four families in which a child had died earlier because of symptoms of the m.3243A > G mutation, so no definite assumption can be made concerning the phenotype of the m.3243A > G mutation in children based on this study. The study does however show that children can be free of symptoms even with a high level of heteroplasmy (up to 93% in UEC). The adult patients with high levels of heteroplasmy developed complaints in late infancy to early adolescence. The younger children with high levels of heteroplasmy should therefore be closely monitored to indentify early symptoms of the clinical expression of the mutation at an early stage.

### MELAS syndrome

The m.3243A > G mutation is most known for its relation with MELAS syndrome. In this study only one patient had stroke-like episodes (SLE) in the past year and three other patients had SLE in the past. Other neurologic symptoms as seizures (n = 9) and encephalopathy (n = 4) also occurred infrequent. Giving an indication that MELAS syndrome is not the primary expression of the m.3243A > G mutation in this cohort. This finding is in line with recent reports (Manwaring et al. 2007) that the m.3243A > G mutation is much more prevalent than previously thought. Patients presenting with symptoms of MELAS syndrome are often referred to a specialized center in which exploratory research for mitochondrial mutation in performed. A patient with the m.3243A > G mutation with a MELAS syndrome phenotype is therefore unlikely to be missed.

### Maternally inherited diabetes deafness (MIDD)

Forty-eight patients (67.6%) of this study had self reported hearing loss. Thirty-seven patients (52.1%) have diabetes mellitus. Only six of the 37 patients with diabetes mellitus do not have hearing loss. Making a total of 31 patients in this study that have diabetes deafness, giving an indication that MIDD is a more frequent expression of the m.3243A > G mutation than MELAS syndrome. The combination of diabetes mellitus and hearing loss is however less a trigger to refer a patient to a specialized center. So a mutation analysis is less frequently performed.

### Multiorgan involvement

Besides the typical symptoms of MELAS syndrome or MIDD we observed high frequencies of other symptoms. Cardiac involvement, gastro-intestinal symptoms and visual disturbances are most frequent. Cardiac involvement has previously been described (Fayssoil 2009; Lev et al. 2004; Wortmann et al. 2007). In our cohort we found a large group of patients with asymptomatic ECG changes on one hand, but some severely affected patients as well. In the four patients with an ejection fraction of less than 30% the cardiac problems dominate the clinical picture. A large number of patients in our cohort exhibits gastro-intestinal problems. In most cases patients suffer from mild to moderate constipation or symptoms of irritable bowel syndrome. In five patients symptoms required admission or even surgical intervention. Cases of chronic intestinal pseudo obstruction have previously been described in patients with the m.3243A > G mutation (Bergamin et al. 2008; Verny et al. 2008). Twenty-six percent of the patients reported visual disturbances and 42% of the patients did not have a vision of 1.0 on a brief visual acuity test. Previous reports indicate the presence of macular dystrophy in patients with the m.3243A > G mutation (Massin et al. 1999; Michaelides et al. 2008). Because of this multi-organ involvement of the m.3243A > G mutation we recommend that all carriers of the mutation are checked for early stages of these symptoms by specialized physicians, including a consultation by a cardiologist and a ophthalmologist, also when there is no diabetes present.

### Quality of life

We assessed quality of life in our cohort using the SF12v2questionnaire. As visualized in Fig. 2, a lower QoL_P_ is negatively correlated to the NMDAS. Indicating, as suspected, that when a patient has more symptoms of the m.3243A > G mutation, a lower QoL_P_ is reported. QQoL_M_ was not different from healthy controls nor from their relatives that do not carry the mutation. Twenty patient did report a history of depression or anxiety. This was however not significantly different from their relatives that did not carry the mutation.

### Different samples

As indicated in Fig. 5a-c we found a correlation between NMDAS and heteroplasmy in all three samples, of which buccal mucosa had the best correlation (r = 0.427, p < 0.001 versus r = 0.254, p = 0.032 in leucocytes and r = 0.294 , p = 0.016 in UEC). When we selected for the most severely affected patients (NMDAS > 20) we found stronger correlation for leucocytes (r = 0.366 , p = 0.072) and UEC (r = 0.530, p = 0.008). Whittaker et al. (2009) was the first to describe a correlation between heteroplasmy and symptoms. We confirm the good ability of UEC heteroplasmy predict clinical outcome but add that this accounts most for the more severely affected patients. In the general group of carriers of the m.3243A > G mutation heteroplasmy in buccal saliva has a better ability to predict clinical outcome.

Different studies have described a decrease in heteroplasmy with increasing age (Frederiksen et al. 2006; Ma et al. 2010; Rahman et al. 2001). As demonstrated in Fig. 4a-c, we also find a decreasing heteroplasmy in all samples. The decrease in heteroplasmy with increasing age is much more prominent in leucocytes (r = -0.705, p < 0.001) than in the other samples (UEC r = -0.374, p = 0.001, buccal mucosa r = -0.460, p < 0.001). However the decrease in heteroplasmy level with age can only be confirmed after a prospective follow-up study. We also found that heteroplasmy in UEC has a 26 percent point higher value than heteroplasmy in leucocytes and a 11 percent point higher value than heteroplasmy in buccal mucosa. The m.3243A > G mutation was undetectable in leucocytes in 12 patients in which the mutation was detected in UEC. Of these 12 patients the mutation was also undetectable in buccal mucosa in seven patients. There was one patient in which the m.3243A > G mutation could not be detected in UEC, where it was detected in leucocytes and buccal mucosa. However we should take into account that we can never predict the values of untested tissues such as muscle, heart, kidney, liver or brain. This means that UEC are the best non-invasively available tissue to test if a patient is suspected of the m.3243A > G mutation.

## Conclusion

We conclude that the m.3243A > G mutation causes a wide variety of signs and symptoms, MIDD being the most prevalent phenotypic expression. Of the three non-invasively available tissues for testing heteroplasmy, we advise to use UEC to detect the mutation. Dormant carriers should be checked regularly by a specialized physician to see whether the disease has awakened and to be early in preventing and treating symptoms.

## Abbreviations

OXPHOS: Oxidative phosphorylation
mtDNA: Mitochondrial DNA
nDNA: Nuclear DNA
UEC: Urinary epithelial cells
MELAS: Mitochondrial myopathy encephalopathy, lactate acidosis and stroke-like episodes
MIDD: Maternally inherited diabetes and deafness
NMDAS: Newcastle Mitochondrial Disease Adult Scale
NPMDS: Newcastle Pediatric Mitochondrial Disease Scale
FSGS: Focal segmental glomerulosclerosis
LVH: Left ventricular hypertrophy
MERRF: Myoclonic epilepsy with ragged-red fibers
dNTP’s: Deoxynucleotide triphosphates
PPi: Pyrophosphate
QoL: Quality of life
QoL_P_: Quality of physical health
QoL_M_: Quality of mental health
